# Imaging-Based Diagnosis of Epiconus Syndrome From the Distance of the Lesion to Where the Spinal Cord Terminates Not From the Disc Level: A Case Series

**DOI:** 10.7759/cureus.17708

**Published:** 2021-09-04

**Authors:** Tomoyuki Asada, Masao Koda, Toru Funayama, Hiroshi Takahashi, Hiroshi Noguchi, Kousei Miura, Kentaro Mataki, Masashi Yamazaki

**Affiliations:** 1 Department of Orthopedic Surgery, Faculty of Medicine, University of Tsukuba, Tsukuba, JPN

**Keywords:** epiconus syndrome, thoracolumbar lesion, differential diagnosis, spinal cord terminus, thoracic myelopathy

## Abstract

Objective

This study aimed to analyze the neurological symptoms caused by thoracolumbar lesions according to their distance from where the spinal cord terminates for a better description of epiconus syndrome.

Methods

We retrospectively reviewed cases of patients with neurological symptoms caused by a thoracolumbar lesion in a single institute. Neurological symptoms were analyzed according to the distance from the proximal end of the lesion to where the spinal cord terminates using MRI or CT myelograms. The symptoms were classified into epiconus syndrome, thoracic myelopathy, and conus medullaris syndrome. The distance was described regarding the length of a vertebral body (VB).

Results

We included 19 patients in this series. The spinal cord terminates were at the lower third of the L1 vertebra most frequently (32%) in the range of T12 to L2 vertebra. The border between thoracic myelopathy and epiconus syndrome was 2VB proximal from where the spinal cord terminates, and that between epiconus syndrome and conus medullaris syndrome was 1VB. Mean disease duration until symptoms changed was 2.4 months in epiconus syndrome, while it was 25 months in thoracic myelopathy, and 10.3 months in conus medullaris syndrome.

Conclusion

Epiconus syndrome is caused by lesion 1-2VB proximal to where the spinal cord terminates. This study may provide further helpful information for clinical practice in the treatment of epiconus syndrome.

## Introduction

The spinal cord at the thoracolumbar junction includes upper and lower neurons because both lumbar to sacral segments and nerve roots exist in a small sagittal length. Symptoms of spinal compression in this region vary from myelopathy to radicular pain. Due to their characterized anatomy, a difference of one vertebral level can lead to a substantial difference in symptoms.

Epiconus syndrome, which is elicited from spinal lesions just proximal to the conus medullaris, is described in previous reports and textbooks highlighting the complexity of the symptoms [1-4]. These symptoms include radicular pain mimicking the symptoms from lower lumbar lesions regardless of their anatomical location [4]. Moreover, these thoracolumbar and lower lumbar lesions often coexist because lumbar lesions are a common pathology [5]. Therefore, the similarity of the symptoms often makes it difficult for spine surgeons to determine the main lesion for surgery to treat lumbar spine disease versus thoracolumbar junction disease.

To clarify the symptoms caused by thoracolumbar junction lesions, some previous studies reported the characteristics of symptoms of single-level disc herniation and single-level ossification of the ligamentum flavum in the thoracolumbar junction [2,3]. However, these studies focused on the disc level, whereas the place where the spinal cord terminates and segment levels differ among individuals [6,7]. Considering each neurological segment at the thoracolumbar junction is located in a narrow space in the spinal cord, the variation in the level at which the spinal cord terminates could have a substantial impact on clinical decision making [2].

To our knowledge, no previous English language report has indicated the relationship between the distance from where the spinal cord terminates and the location of symptomatic characteristics of thoracolumbar lesions. We hypothesized that the border of syndromes caused by thoracolumbar lesions would be illustrated better by focusing on the distance from the lesion to where the spinal cord terminates than from the disc level. Thus, the purpose of this study was to analyze the symptoms caused by thoracolumbar lesions and to classify them according to their distance from where the spinal cord terminates.

## Materials and methods

Study population

After approval of the present study by our institutional review board, medical records in the electronic medical system at our institution were reviewed for this clinical case series. Written informed consent to involve this study was obtained from all individual participants. Inclusion criteria were patient records with the following: (1) the diagnostic name in regards to the thoracolumbar lesion, (2) magnetic resonance imaging (MRI) or computed tomography (CT) indicating the thoracolumbar spinal lesion, (3) descriptions of neurological symptoms from the suspected thoracolumbar compressive lesion before surgery, and (4) surgery successfully improving the neurological symptoms. A thoracolumbar lesion was defined as a spinal cord compressive lesion from the T10-11 disc level to the L1-2 disc level [3]. As for patients with multiple compressive lesion from thoracic to lumbar spine, the most proximal end was recorded as the causative lesion. Exclusion criteria were (1) patients without follow-up, (2) those with the diagnostic name of cauda equina syndrome in the medical record, and (3) those who we grouped into cauda equina syndrome (see subsection - Grouping patients according to symptoms).

From the medical records, the following variables were reviewed: age, sex, initial diagnosis at the primary hospital, final diagnosis at our hospital, delay of diagnosis, initial symptoms, chief complaint requiring surgery, the interval between initial symptom and surgery, spinal cord termination, a proximal end of the lesion, the physical examination, and the types of surgery. The initial diagnosis was recorded from the referral letter. A physical examination finding included leg pain assessed with a visual analog scale (VAS), lower back pain (LBP), manual muscle testing (MMT), deep tendon reflex (DTR), bowel and bladder dysfunction (BBD), and Babinski reflex. BBD included frequent urination, residual urination, urinary retention, and incontinence. A delayed diagnosis was defined as a case in which a patient was referred to our hospital after receiving a different diagnosis at another hospital, and the patient received a different diagnosis from the previous one at our hospital, and surgery was performed for the new diagnosis. The lower lumbar disease was defined as a radiological lesion below the L2-3 disc level.

Radiological examination

**Figure 1:**
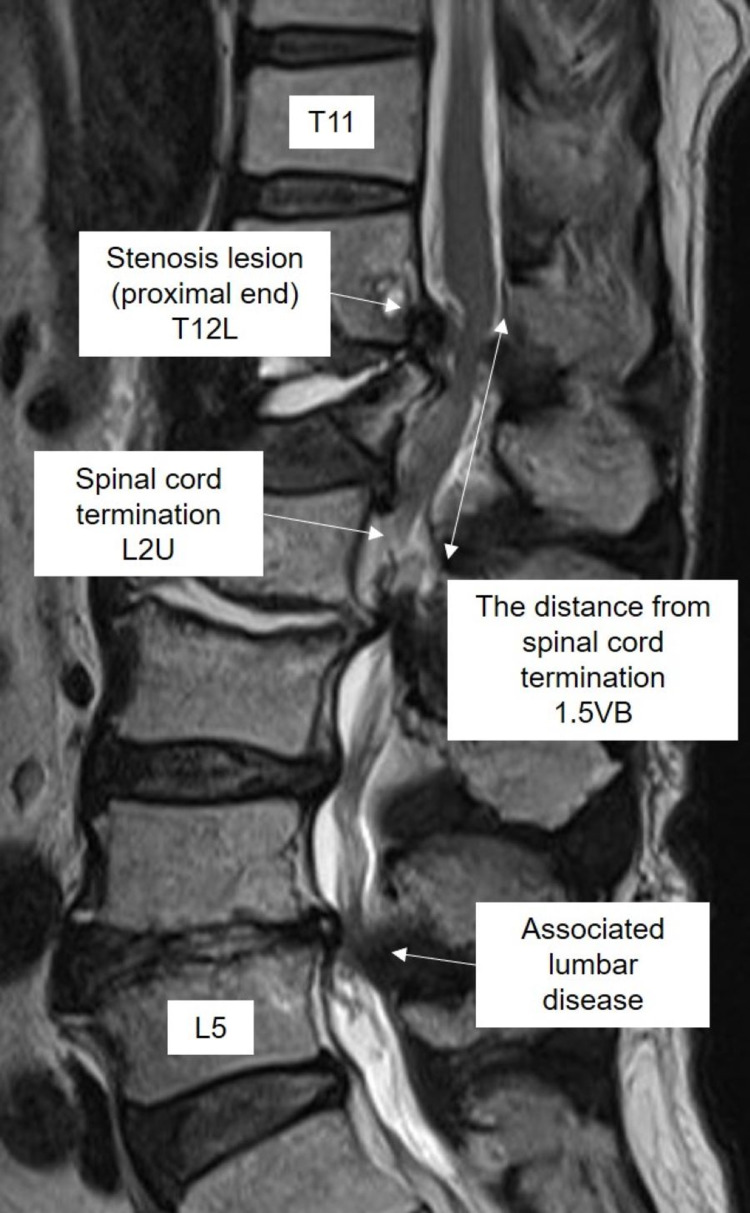
Measurement of the distance of the lesion from where the spinal cord terminates U: upper third of vertebral body; L: lower third of vertebral body; VB: vertebral body

MRI or CT myelograms before the surgery were assessed as radiological data. The level at which the spinal cord terminates was based on the images before surgery. The distance from the spinal cord termination was defined as the distance from the proximal end of the lesion. The distance in the spinal canal was measured using a method reported previously [4]. Each vertebral level consists of three segments of the vertebral body (upper, U; middle, M; lower, L), and each weighted at 0.25 vertebral body (VB), as well as a disc weighted as 0.25VB. In short, 1VB equals 0.25VB(U) + 0.25VB(M) + 0.25VB(L) + 0.25VB(D). For example, Figure 1 shows that the spinal cord terminates at L2U, the proximal end of the lesion was T12L, and the distance of the lesion from where the spinal cord terminates was 1.5VB, which consists of L1 body (0.75VB) + L1/2D (0.25VB) + L2U (0.25VB). If the lesion was so severe as to obscure where the spinal cord terminates before the surgery, the level was confirmed with follow-up MRI after surgery.

Grouping patients according to symptoms

Thoracolumbar spinal lesions can cause the following four syndromes: thoracic myelopathy, epiconus syndrome, conus medullaris syndrome, and cauda equina syndrome. Based on previous reports, the key symptoms of each syndrome were defined as follows: myelopathy with “hyperreflexia in the lower leg,” epiconus syndrome with “paresis, radicular leg pain and decreased reflex,” conus medullaris syndrome with “BBD before severe leg pain or paralysis,” and cauda equina syndrome with "bilateral multiple radicular pain aggravated by walking" [3,4,8-10]. Based on these symptoms, we diagnosed each patient with the four symptoms above. As for cauda equina syndrome, since there is a wide range in the definition of symptoms, the stenosis level should be taken into account, and the presence of a proximal stenotic lesion distal to the spinal cord termination (SCT) in addition to the key symptoms was the basis for the diagnosis. The patients with mild neurological symptoms (e.g., if leg pain was the only symptom throughout the entire physical examination) were grouped into those with the same syndrome as that for the same or adjacent injured level.

## Results

Of the 19 patients included in this series, nine were female. The final diagnosis of each patient was as follows: disc herniation (DH), one; ossification of ligamentum flavum (OLF), one; schwannoma, nine; fracture, four; tumor other than schwannoma, two; intradural hemorrhage, two. Two cases with hemorrhage were diagnosed as acute hemorrhage from intradural hemangioma. Lumbar lesions other than thoracolumbar lesions were associated with 10 cases (47%). A delayed diagnosis was found in nine cases (42%) (Table 1). All surgery successfully improved the symptoms of the patients.

**Table 1:** Diagnosis and physical examination No.: case number; PTR: patellar tendon reflex; ATR: Achilles tendon reflex; IP: iliopsoas muscle; Q: quadriceps muscle; TA: tibial anterior muscle; EHL: extensor hallucis longus muscle; GC: gastrocnemius muscle; BBD: bladder and bowel dysfunction; Rt: right leg; Lt: Left leg; Bi: bilateral; OLF: ossification of ligamentum flavum; DH: disc herniation; OVF: osteoporotic vertebral fracture; NA: not available in electronic medical system; M: male; F: female

Case	Age	Sex	Final diagnosis	Lower lumbar lesion	Delayed diagnosis	Distance from SCT	PTR		ATR		IP		Q		TA		EHL		GC		BBD	Leg pain		VAS	Syndromes
							Rt	Lt	Rt	Lt	Rt	Lt	Rt	Lt	Rt	Lt	Rt	Lt	Rt	Lt					
1	76	M	OLF	-	No	2.5	2	2	2	2	4	4	3	3	0	0	0	0	0	0	incontinence	Bi	anterior thigh	0	myelopathy
2	46	F	schwannoma	+	Delayed	2.25	2	3	2	3	5	5	5	5	5	5	5	5	5	5	none	Lt	medial whole leg	7	myelopathy
3	37	M	schwannoma	-	No	2	2	2	0	0	5	5	5	5	5	5	5	5	5	5	none	Rt	anterior thigh	3	myelopathy
4	70	F	schwannoma	+	Delayed	2	0	0	2	2	5	5	5	5	5	5	5	5	5	5	none	Rt	posterior whole leg	5	myelopathy
5	47	M	DH	-	No	2	2	2	2	2	4	5	3	4	5	5	5	5	5	5	frequency	Bi	whole leg	5	myelopathy
6	78	F	OVF	+	No	2	0	0	1	1	1	4	1	4	5	5	5	5	5	5	none	Bi	anterior thigh	5	epiconus syndrome
7	63	M	schwannoma	+	Delayed	1.5	2	2	2	2	5	5	5	5	5	5	5	5	5	5	none	Lt	anterior thigh	1	myelopathy
8	75	M	OVF	+	No	1.5	1	1	0	0	5	5	5	5	3	3	3	3	5	5	none	Bi	anterior thigh	3	epiconus syndrome
9	75	M	OVF	+	No	1.5	2	0	0	0	5	5	5	5	2	3	2	3	3	3	retention	Bi	anterior lower leg	3	epiconus syndrome
10	74	F	schwannoma	-	No	1.5	1	1	0	0	5	5	5	5	2	2	2	2	2	2	none	Lt	anterior thigh	5	epiconus syndrome
11	82	M	OVF	+	Delayed	1.25	0	0	0	0	5	5	5	5	5	5	5	5	5	5	none	Rt	lateral thigh	8	epiconus syndrome
12	54	F	schwannoma	-	No	1.25	1	1	1	1	5	5	5	5	5	5	5	5	5	5	none	Rt	posterior thigh and latelra lower leg	7	epiconus syndrome
13	71	M	hemorrhage	+	No	1	0	0	1	1	4	5	4	5	2	5	2	4	4	5	none	Rt	posterior thigh and lower leg	8	epiconus syndrome
14	77	M	schwannoma	-	No	0.75	0	0	0	0	5	5	5	5	5	5	5	5	5	5	frequency	Lt	anterior thigh	4	conus medullaris syndrome
15	75	F	schwannoma	+	Delayed	0.5	1	1	0	0	5	5	5	5	5	5	5	5	5	5	none	Rt	lateral thigh	6	conus medullaris syndrome
16	78	M	tumor other than schwannoma	-	Delayed	0.5	NA	NA	NA	NA	5	5	5	5	5	5	5	5	5	5	retention	Rt	posterior whole leg	7	conus medullaris syndrome
17	65	M	schwannoma	+	Delayed	0.25	2	2	1	1	4	4	4	4	4	4	4	4	4	4	none	Bi	posterior whole leg	4	conus medullaris syndrome
18	33	F	tumor other than schwannoma	-	Delayed	0	0	0	0	0	5	5	5	5	5	5	5	5	5	5	none	Lt	anterior thigh and lower leg	0	conus medullaris syndrome
19	69	F	hemorrhage	-	No	-0.25	3	3	1	1	5	5	5	5	5	5	5	5	5	5	frequency	Rt	posterior thigh and lateral lower leg	7	conus medullaris syndrome

**Figure 2:**
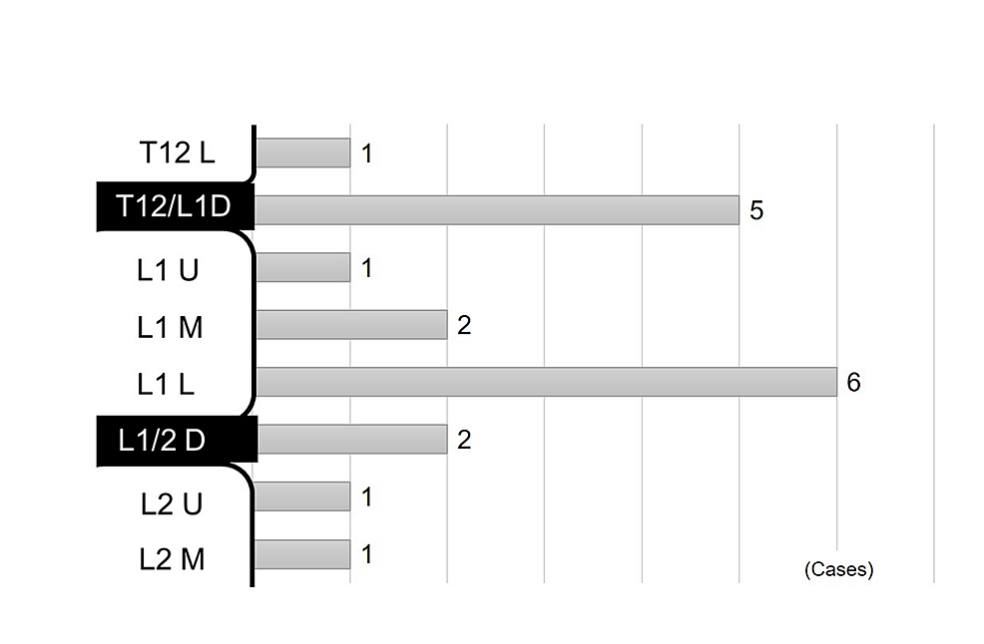
Distribution of the level at which the spinal cord terminates U: upper third of vertebral body; M: middle third of vertebral body; L: lower third of vertebral body; D: vertebral disc

**Figure 3:**
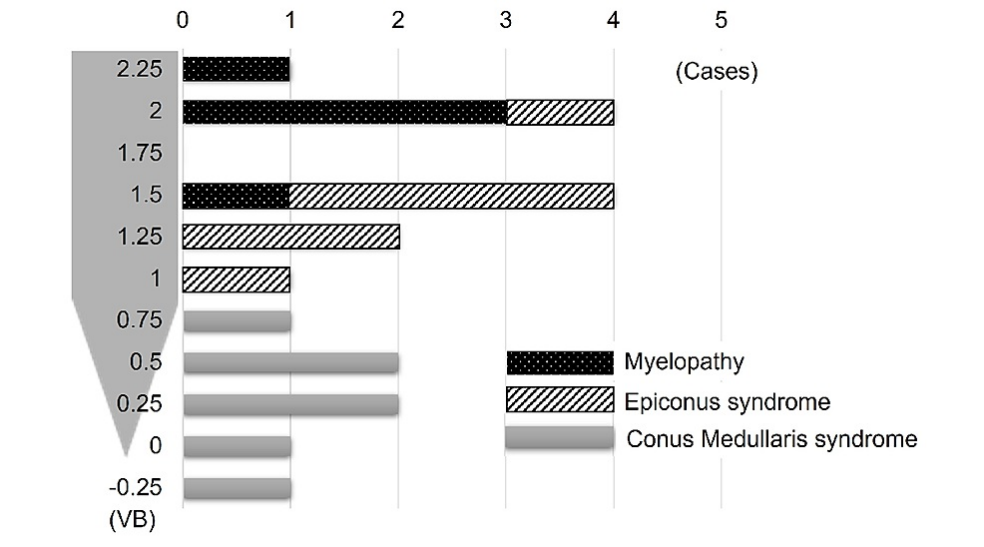
Distance of the lesion from where the spinal cord terminates VB: vertebral body

L1L was the most common level at which the spinal cord terminates (n = 6/19, 32%) in the range of T12L to L2M (Figure 2). The distance from the lesion to where the spinal cord terminates varied from -0.25 to +2.5 VB (Figure 3). 

Six patients showed symptoms of myelopathy (Table 2). The distance from the lesion ranged from +2.5 to 1.5VB proximal to where the spinal cord terminates. The average interval between initial symptoms and surgery was 24 months (Table 2). Three patients (cases 2, 4, and 5) experienced worsened initial symptoms for more than one year, and two patients (cases 1, and 5) had mild BBD, which did not need emergency surgical intervention. The patient with case 7 showed hyperreflexia of both PTR and ATR, interpreted as myelopathy regardless of the distance from the lesion to where the spinal cord terminates.

**Table 2:** Patients with myelopathy No.: case number; VB: vertebral body; SCT: spinal cord termination; Th11M: middle third of Th11 vertebral body; Th11L: lower third of Th11 vertebral body; L1M: middle third of L1 vertebral body; L1L: lower third of  L1 vertebral body; L2M: middle third of L2 vertebral body; Th10/11D: vertebral disc level of Th10/11; Th12/L1D: vertebral disc level of Th12/L1; PDF: posterior decompression and fusion; LBP: lower back pain; M: male; F: female

No.	Sex	Age	Initial symptom	Chief complaint requiring surgery	Initial symptom to surgery (months)	SCT	Stenosis lesion	Distance from SCT (VB)	Surgery
1	M	76	gait disturbance	gait disturbance	48	L1M	Th10/11D	2.5	PDF
2	F	46	paresthesia in both lower legs	worsened paresthesia	27	L1L	Th11M	2.25	tumor resection
3	M	37	LBP	LBP	5	L1M	Th11M	2	tumor resection
4	F	70	LBP, paresthesia in Rt. lower leg	worsened paresthesia	36	L1M	Th11M	2	tumor resection
5	M	47	gait disturbance	gait disturbance	28	L1L	Th11L	2	anterior herniotomy
7	M	63	LBP, Lt. leg pain	motor weakness	4	L2M	Th12/L1D	1.5	tumor resection

Of the seven patients with epiconus syndrome, the distance from the lesion was from +1 to +2VB proximal to where the spinal cord terminates. Five patients (71%) required surgery because of motor weakness and gait disturbance. The interval between their initial symptoms and surgery was nine months (Table 3). The patient with case 6 had no hyperreflexia with proximal lower leg muscle weakness, interpreted as the epiconus syndrome rather than myelopathy.

**Table 3:** Patients with epiconus syndrome SCT: spinal cord termination; VB: vertebral body; Th11M: middle third of Th11 vertebral body; Th11L: lower third of Th11 vertebral body; Th12U: upper third of Th12 vertebral body; Th12L: lower third of Th12 vertebral body; L1L: lower third of  L1 vertebral body; L2U: upper third of L2 vertebral body; Th11/12D: vertebral disc level of Th11/12; Th12/L1D: vertebral disc level of Th12/L1; L1/2D: vertebral disc level of L1/2; BKP: balloon kyphoplasty; LBP: lower back pain; M: male; F: female

No.	Sex	Age	Initial symptom	Chief complaint requiring surgery	Initial symptom to surgery (months)	SCT	Stenosis lesion	Distance from SCT (VB)	Surgery
6	F	78	LBP gait disturbance	motor weakness	14	L1/2D	Th11/12D	2	posterior fusion
8	M	75	LBP	motor weakness	2	L1L	Th12U	1.5	BKP+laminectomy
9	M	75	gait disturbance	gait disturbance	3	L1L	Th12U	1.5	anterior fusion
10	F	74	gait disturbance	gait disturbance	8	Th12/L1D	Th11M	1.5	tumor resection
11	M	82	LBP	LBP	7	L2U	Th12L	1.25	anterior fusion
12	F	54	gait disturbance	worsened pain in leg	24	Th12/L1D	Th11L	1.25	tumor resection
13	M	71	both leg pain	motor weakness	4	L1/2D	Th12/L1D	1	tumor resection

Of six patients with conus medullaris syndrome, LBP (cases 14, 15, and 18) or leg pain (cases 17, and 19) was the main reason for surgery (Table 4). The patient with case 16 experienced BBD without lower leg paresis. Two patients (cases 17 and 19) exhibited unilateral hyperreflexia and the patient with case 17 exhibited mild whole leg paresis. These two patients were interpreted as having conus medullaris syndrome because of the level of the lesion, despite the results of the physical examination.

**Table 4:** Patients with conus medullaris syndrome SCT: spinal cord termination; Th12U: upper third of Th12 vertebral body; Th12L: lower third of Th12 vertebral body; L1U: upper third of L1 vertebral body; L1L: lower third of  L1 vertebral body; Th12/L1D: vertebral disc level of Th12/L1; LBP: lower back pain; BBD: bladder and bowel dysfunction; M: male; F: female

No.	Sex	Age	Initial symptom	Chief complaint requiring surgery	Initial symptom to surgery (months)	SCT	Stenosis lesion	Distance from SCT (VB)	Surgery
14	77	M	LBP	LBP	10	Th12/L1D	Th12U	0.75	tumor resection
15	75	F	LBP	worsened LBP	15	Th12L	Th12U	0.5	tumor resection
16	78	M	urinary dysfunction	BBD	0.5	L1L	L1U	0.5	tumor resection
17	65	M	both leg pain	worsened leg pain	52	Th12/L1D	Th12L	0.25	tumor resection
18	33	F	LBP gait disturbance	worsened LBP	6	L1U	L1U	0	tumor resection
19	69	F	Rt. leg pain	Rt. leg pain	48	Th12/L1D	L1U	-0.25	tumor resection

From the results mentioned above, the clinical features of each syndrome were summarized in Table 5. A distance from the lesion from 1.5-2VB to 0.75-1VB to where the spinal cord terminates could be considered as the border of myelopathy to epiconus syndrome, and epiconus syndrome to conus medullaris syndrome, respectively. Thus, we classified patients into three groups with borders of 1VB and 2VB to simplify them. Based on Table 5, a clinical diagnostic chart was developed by focusing on the distance from the lesion to where the spinal cord terminates to characterize each syndrome (Table 6).

**Table 5:** Summary of each syndrome Patients’ numbers with each symptom or physical findings are listed in the columns below initial symptoms. SCT: spinal cord termination; VB: vertebral body; LBP: lower back pain; BBD: bladder and bowel dysfunction; IP: iliopsoas muscle; Q: quadriceps muscle; TA: tibial anterior muscle; EHL: extensor hallucis longus muscle; GC: gastrocnemius muscle; PTR: patellar tendon reflex; ATR: Achilles tendon reflex; BBD: bladder and bowel dysfunction

Syndrome		Myelopathy	Epiconus	Conus medullaris
The distance from SCT (VB)		1.5 to 2.5	1 to 2	-0.25 to 0.75
Average time between initial symptom to surgery (months)		24	9	22
Initial symptoms	Paresthesia or leg pain	2	0	3
	LBP	2	3	1
	Gait disturbance	2	3	1
Symptom requiring surgery	Paresthesia or leg pain	2	1	2
	Gait disturbance	2	2	0
	Paresis	1	3	0
	LBP	1	1	3
	Other	0	0	BBD, 1
Paralyzed muscle	IP, Q	3	1	1
	TA, EHL	1	4	1
	GC	1	3	1
PTR/ATR		↑/↑	↓/↓	↓or↑/↓
BBD		3	1	3
Babinski reflex(es)		1	0	0

**Table 6:** Diagnostic chart of thoracolumbar lesion SCT: spinal cord termination; VB: vertebral body; IP: iliopsoas muscle; Q: quadriceps muscle; TA: tibial anterior muscle; EHL: extensor hallucis longus muscle; GC: gastrocnemius muscle; PTR: patellar tendon reflex; BBD: bladder and bowel dysfunction

Syndrome	Myelopathy	Epiconus	Conus medullaris
The distance from SCT (VB)	>2	1–2	<1
Initial symptom to surgery (months)	> 1 year	<1year	
Paresthesia or leg pain	++	+	++
Leg pain	+	+	++
Motor weakness	++	+++	-
Paralyzed muscle	IP, Q	TA, EHL	-
PTR	↑	↓	↓ or ↑
BBD	+	-	++

## Discussion

This retrospective case series reviewed 19 cases of thoracolumbar lesions requiring surgery. Some 47% were associated with lumbar disease and diagnosis was delayed in 43% of patients. This indicated that the neurological level of diagnosis in thoracolumbar lesions remains challenging as previously reported [1,10-13]. More, the symptomatic similarity of epiconus syndrome with the lumbar disease was not still under-recognition even in orthopedic surgeons although the differential diagnosis is important. To further understand the syndromes and facilitate their diagnosis, we recommend the diagnostic chart according to the distance from the lesion to where the spinal cord terminates based on present findings (Table 6). As a result of this study, epiconus syndrome can be induced from the lesion between 1 and 2VB proximal to where the spinal cord terminates, which possibly has the potential to indicate faster operative treatment rather than myelopathy.

A study of cadavers indicated that the vertebral level at which the spinal cord terminates has a wide variation distributed from T12 to the lower third of L3, possibly resulting in difficulties to determine the main lesion based on vertebral level [14]. However, other studies analyzed thoracolumbar syndromes according to the vertebral levels, not the distribution of spinal cord segments [2,3,15]. Anatomical studies revealed that the S2-S5 segment was located at 34.9 mm and L4-S1 segment length was 60.5-34.9 mm proximal to where the spinal cord terminates, which indicates the segment location should be decided using the distance from where the spinal cord terminates, and not from the vertebral level [1-3,6]. The present findings indicated that the level at which the spinal cord terminates ranges from T12L to L2M, which is consistent with the variation found by previous studies. Thus, the main lesion could be determined not by the vertebral level, but by the distance from the lesion to where the spinal cord terminates. Analysis of the distance from the thoracolumbar lesion to where the spinal cord terminates gives us a new perspective for making a precise diagnosis in patients with various pathologies, especially for those with epiconus syndrome whose diagnosis is difficult.

The present results revealed that the border between myelopathy and epiconus syndrome was 1.5-2VB from where the spinal cord terminates, as discriminated by hyperreflexia. Together with a previous report, which indicated that epiconus was located at 1.6 ± 0.4VB proximal to where the spinal cord terminates, 1.5-2VB from where the spinal cord terminates might be considered as a reasonable border between myelopathy and epiconus syndrome, although this remains controversial [4].

The symptomatic difference between epiconus syndrome and conus medullaris syndrome was set with the presence of BBD. Typical conus medullaris syndrome, which presented BBD without any other neurological findings, was found in only one patient (case 16). A typical conus medullaris syndrome is relatively rare because of the transitional anatomy of the thoracolumbar lesion [10]. The most common initial complaint is LBP and leg pain, as distinct from the symptoms of patients with epiconus syndrome. The border between epiconus syndrome and conus medullaris syndrome was assumed to be 0.75-1VB proximal to where the spinal cord terminates, as judged by the symptomatic character of the patients. Because the mean S2-S5 segmental length is 34.9 mm and that of the vertebral body is around 30 mm, which is consistent with the assumption from the present case series, that the S2-S5 segment is located at 1VB from where the spinal cord terminates, appears reasonable [2,6].

In addition, one of the possible clinical characteristics of epiconus syndrome obtained from the present case series is the rapid progression of motor weakness. The mean duration of disease, until symptoms changed, was 2.4 months in epiconus syndrome, while it was 10.3 months in conus medullaris syndrome and 25 months in myelopathy, suggesting more rapid progression of epiconus syndrome than that of conus medullaris syndrome and thoracic myelopathy. Although the exact mechanism underlying the rapid progression of epiconus syndrome remains unclear, direct compression of motor neurons located in the anterior horn of spinal cord gray matter might lead to a rapid progression of motor weakness. For a better surgical outcome, early diagnosis and surgical intervention before the motor weakness progress are theoretically essential.

Limitation

Some limitations should be addressed. First, this study is a retrospective case series with a small sample number. Recall bias in the clinical course cannot be denied. More cases of thoracolumbar lesions are needed to confirm clinical data. Second, all of the patients included in this case series were Japanese and where the spinal cord terminates may be different in patients with other ancestries; therefore, an analysis of epiconus syndrome by focusing on the distance from where the spinal cord terminates is warranted for those patients who are not Japanese [7]. The third is the biased distribution of the syndromes; 11 of 19 patients had a spinal tumor. The traits of tumors could have some impact on the clinical course of the patients. Last, cases treated conservatively were not included in this study. It remains unclear how severe cases with a herniated disc or vertebral fracture need to be to warrant surgical intervention. Further studies of cases with this lesion are needed to resolve these issues.

## Conclusions

Although the differential diagnosis between lower lumbar lesions and thoracolumbar lesions remains challenging, the present data obtained from neurological and radiological analyses suggest that epiconus syndrome is caused by a lesion 1-2VB proximal to where the spinal cord terminates and shows the relatively rapid progression of paralysis.
